# Effects and related mechanism of alpha-adrenergic receptor inhibitor phentolamine in a rabbit model of acute pulmonary embolism combined with shock

**DOI:** 10.1186/s40001-022-00842-5

**Published:** 2022-11-08

**Authors:** Yuting Wang, Li Qiu, Delong Yu, Yijun Yu, Liqun Hu, Ye Gu

**Affiliations:** Department of Cardiology, Wuhan Fourth Hospital, Puai Hospital Affiliated to Tongji Medical College, Huazhong University of Science and Technology, HanZheng Street 473# QiaoKou District, Wuhan, 430033 China

**Keywords:** Acute pulmonary embolism, Sympathetic activity, Alpha receptors, Pulmonary vasoconstriction, Attenuation of pulmonary blood flow

## Abstract

**Background:**

To observe the effect and mechanism of alpha-adrenergic receptor inhibitor phentolamine (PTL) in a rabbit model of acute pulmonary embolism (APE) combined with shock.

**Methods:**

Twenty-four New Zealand rabbits were randomly divided into sham operation group (S group, *n* = 8), model group (M group, *n* = 8) and PTL group (*n* = 8), the model of APE combined with shock was established. Mean pulmonary arterial pressure (MPAP), peripheral mean arterial pressure (MAP) and pulmonary circulation time were evaluated. The expression levels of α_1_ receptor, α_2_ receptor and their downstream molecules in pulmonary embolism (PE) and non-pulmonary embolism (non-PE) regions lung tissues were detected and compared, respectively.

**Results:**

In M group, α receptor-related signaling pathways were significantly activated in both PE and non-PE areas as expressed by up-regulated α_1_, α_2_ receptor and phospholipase C (PLC); the expression level of phosphorylated protein kinase A (p-PKA) was significantly down-regulated; myosin light chain kinase (MLCK) and α-smooth muscle actin (α-SMA) levels were up-regulated. PTL treatment significantly improved pulmonary as well as systemic circulation failure: decreased MPAP, restored blood flow in non-PE area, shortened pulmonary circulation time, increased MAP, and restored the circulation failure. PTL induced significantly down-regulated expression of α_1_ receptor and its downstream molecule PLC in both PE and non-PE area, the expression level of α_2_ receptor was also down-regulated, the expression level of p-PKA was significantly up-regulated. PTL treatment can inhibit both α_1_ and α_2_ receptor-related signaling pathways in whole lung tissues, and inhibit Ca^2+^ signaling pathways. The expression level of MLCK and α-SMA were significantly down-regulated. Compared with PE area, the changes of expression levels of α receptor and its downstream molecules were more significant in the non-PE region.

**Conclusion:**

In this model of APE combined with shock, the sympathetic nerve activity was enhanced in the whole lung, α_1_ and α_2_ receptor and their downstream signaling activation might mediate blood flow failure in the whole lung. PTL treatment can effectively restore pulmonary blood flow in non-PE area and improve pulmonary as well as systemic circulation failure possibly through down-regulating α_1_ and α_2_ receptor and their downstream signaling pathways.

**Supplementary Information:**

The online version contains supplementary material available at 10.1186/s40001-022-00842-5.

## Background

Acute pulmonary embolism (APE) is a severe clinical disease related with high mortality [1, 2]. It is known that APE induced by obstruction of a small percentage of pulmonary vascular trees can cause marked reduction of whole pulmonary blood flow [3], which suggests that non-embolized lung tissues are also involved in mediating the failure of pulmonary blood flow in APE. Our previous study showed that hyperactivation of sympathetic nerve and the sharp decline of pulmonary blood flow in the non-embolized lung tissue served as important factors contributing to hemodynamic collapse of pulmonary circulation and systemic circulation in rabbit APE model combined with shock [4–6]. It is well known that tyrosine hydroxylase (TH) catalyzes the synthesis of norepinephrine (NE); the latter can regulate pulmonary vessels tone via stimulating α receptor on the vascular smooth muscle cells. Previous studies have shown that α receptor can modulate Ca^2+^ concentration in pulmonary smooth muscle cells and regulate pulmonary vessels tone through activating its downstream signaling molecule phospholipase C (PLC), etc. [7–9]. Our previous experiment showed that the level of TH in non-pulmonary embolism (non-PE) tissue was significantly increased, and sympathetic nerve activation might thus result in the pulmonary blood flow attenuation in non-PE lung tissue [4]_._ However, the specific mechanism in which how the pulmonary artery smooth muscle contraction is induced by sympathetic nerve activation and whether sympathetic nerve works through α receptor and its downstream signaling pathways is not fully clear. It remains also unknown whether inhibition of α receptor activity could dilate the lung vessel in non-PE tissues and improve the pulmonary blood flow in APE combined with shock. Previous studies demonstrated that alpha-receptor blocker phentolamine (PTL) could alleviate electrical stimulation or norepinephrine-induced vasoconstriction when applied to isolated pulmonary arteries [10, 11]. To our best knowledge, at present, the in vivo efficacy and safety of PTL, as well as its impact on the expression of α receptor are not explored in the rabbit model of APE combined with shock.

To fill above knowledge gaps, firstly, we explored the changes of α receptor (α_1_ and α_2_ receptor) and the downstream signaling molecules such as PLC, protein kinase A (PKA) and myosin light chain kinase (MLCK) in the lungs from the rabbit model of APE combined with shock. Secondly, we evaluated the effectiveness and safety as well as related mechanisms of intra-pulmonary artery administration of PTL in the rabbit model of APE combined with shock.

## Methods

### Animals and anesthesia

All animals and experimental procedures are complied with the Guide for the Care and Use of Laboratory Animals published by the US National Institute of Health [12], and approved by the Animal Care Committee at Huazhong University of Science and Technology. Healthy adult New Zealand rabbits, weighting 2.5–3.0 kg, were purchased from the Experimental Animal Center of Tongji Medical College, Huazhong University of Science and Technology. The rabbits were housed under standard conditions with free access to food and drinking water for 1 week before the experiments.

All rabbits were fasted food and water for the preoperative experiments, and were anesthetized with the 3% sodium pentobarbital anesthesia (20 mg/kg) through the ear vein. After the anesthetization, the rabbit was fixed on the operating table with the supine position and connected to the cardiogram monitor (LEAD-7000, Sichuan Jinjiang Electronic Technology Co. China) [4–6].

### Catheterization and the model establishment

The rabbit skin of right groin area was prepared and disinfected, which the femoral artery and femoral vein were isolated. The Seldinger puncturing method was used to puncture the femoral artery and the femoral vein, which 5F sheath tubes (Radio focus TERUMO) were placed, respectively. Under X perspective, the 4F Cordis sheath was inserted into to the main trunk of the pulmonary artery for real-time monitor of pulmonary artery for real-time monitor of mean pulmonary arterial pressure (MPAP) [4–6]. Mean arterial pressure (MAP) was detected by femoral artery sheath.

APE model was established and autologous blood clots were prepared according to the methods described in our previous studies [4–6]. Briefly, a plastic tube with an inner diameter of 4 mm and length of 20 cm was used to collect 5 mL of autologous venous blood via the femoral vein sheath. The tube was positioned upright and placed into an incubator at 37℃ for 40 min. The diameter and length of the blood clots were measured using a Vernier caliper. The blood clots were then cut into 3 (± 0.5) × 10-mm columns. The clots were then injected through the 4 French catheter preplaced in the main pulmonary artery in a two-stage manner. In the first stage, totally four clots were injected (one by one for 4 times at 60-s interval). The peripheral artery pressure and pulmonary artery pressure (PAP) were monitored during this period. Modeling was considered successful when MAP and MPAP both reached the following criteria: MAP was below 60 mmHg and MPAP increased up to twofold the baseline level and stable for 2 min. If MAP and MPAP did not reach the defined levels after four clot injections (within 15 min after the first clot injection), more clots were injected at 60-s interval in the second stage, and all rabbits reached the modeling criteria in this experiment (mean number of injected clots, 4.9 ± 1.2).

### Grouping and drug administration

Twenty-four rabbits were divided into the sham operation (S group, followed by bolus injection of saline, *n* = 8, puncture and catheterization, followed by bolus injection of saline), the model of APE combined with shock (M group, followed by bolus injection of saline, *n* = 8, puncture and catheterization, bolus injection of autologous blood clots till reaching the shock status, followed by bolus injection of saline), and PTL group (PTL group, *n* = 8, puncture and catheterization, bolus injection of autologous blood clots till reaching the shock status, followed by intra-pulmonary artery ‘bolus’ injection of PTL at the doses of 0.4 mg/kg).

### Hemodynamic monitoring

LEAD-7000 monitor (Sichuan Jinjiang Electronic Technology Co. China) was used for constantly monitoring the blood pressure. MPAP and MAP were collected before embolization, immediately after successful modeling and 15, 30, 60 and 120 min after the moment of successful modeling.

### Pulmonary artery angiography

Pulmonary arterial blood flow imaging was obtained by pulmonary artery angiography (AlluraXper FD20 Philips). 5–7 ml of contrast was injected for the pulmonary angiography with hand injection with a syringe. The pulmonary circulation time was measured as previously described, by calculating the time taken for the contrast medium to reach from the initiation site of the pulmonary artery trunk to the left atrium through the pulmonary artery trunk and branches, pulmonary capillary, and pulmonary vein. The pulmonary circulation time was calculated for all of the survived rabbits at 120 min after the moment of successfully modeling, which was designed as the study end [6].

### Pathological histological examinations

At the end of experiment, the rabbit PE and non-PE tissues were fixed and cut into sections for pathological examination after paraffin embedding, respectively. Immunohistochemistry staining was made for analyzing the following antibodies: α_1_ receptor (1:200, abcam, Cambridge, Massachusetts), α_2_ receptor (1:200, Immunoway), PLC(1:200, abcam), MLCK (1:100, abcam), total protein kinase A (t-PKA) (1:200, Immunoway), p-PKA(1:200, Immunoway), α-SMA(1:200, abcam).

### Western blotting

Total proteins of lung tissues were extracted and determined by BCA method, which then was separated via SDS-PAGE and electro-transferred onto PVDF membranes. After blocking, membranes were incubated with primary antibodies: anti-α_1_ receptor (1:500, abcam, Cambridge, Massachusetts), anti-α_2_ receptor (1:500, Immunoway), anti-PLC (1:1000, abcam), anti-MLCK (1:1000, abcam), anti-t-PKA (1:1000, Immunoway) and p-PKA polyclonal antibodies (1:1000, Immunoway). Post-TBST washing, the secondary antibody such as goat-anti-rabbit, goat-anti-mouse (KPL, USA) were employed for incubation, and the chemical luminescence method was performed to obtain protein band images for the semi-quantitative analysis (Bio-Rad, USA).

### Statistical analysis

All statistical data are expressed as mean ± SD (IBM SPSS 19.0 software). All data were first evaluated for normal distribution using Shapiro–Wilk test. One-way ANOVA followed by Tukey’s or Games–Howell’s post hoc test was used to evaluate the differences among groups. The mortality rate in S, M and PTL groups were compared by log rank test of Kaplan–Meier curves. The value of *P* < 0.05 was considered statistically significant.

## Results

### PTL dose-finding

In the dose-finding phase of the pilot study, the impact of PTL on systemic blood pressure was observed in this APE combined with shock model (as shown in Additional file 1: Figure S1). In sham rabbits, the systemic MAP and heart rate were not affected by intra-pulmonary artery bolus injection of PTL at dosage ranged from 0.1 to 0.5 mg/kg. The systemic MAP and heart rate decreased about 10 mmHg and 30 beats/min, respectively, by PTL at dosage ranged from 0.5 to 1.0 mg/kg. However, MPAP was not affected by PTL at above dosage.

In APE model combined with shock, the systemic MAP and heart rate tended to increase, while MPAP decreased significantly by PTL at dosage ranged from 0.1 to 0.4 mg/kg. Systemic MAP and heart rate tended to decrease, while MPAP remained unchanged (Additional file 2) at PTL dosage ranged from 0.5 to 1.0 mg/kg. Therefore, PTL at the dosage of 0.4 mg/kg was used in the main experiment.

### Survival results

**Fig. 1:**
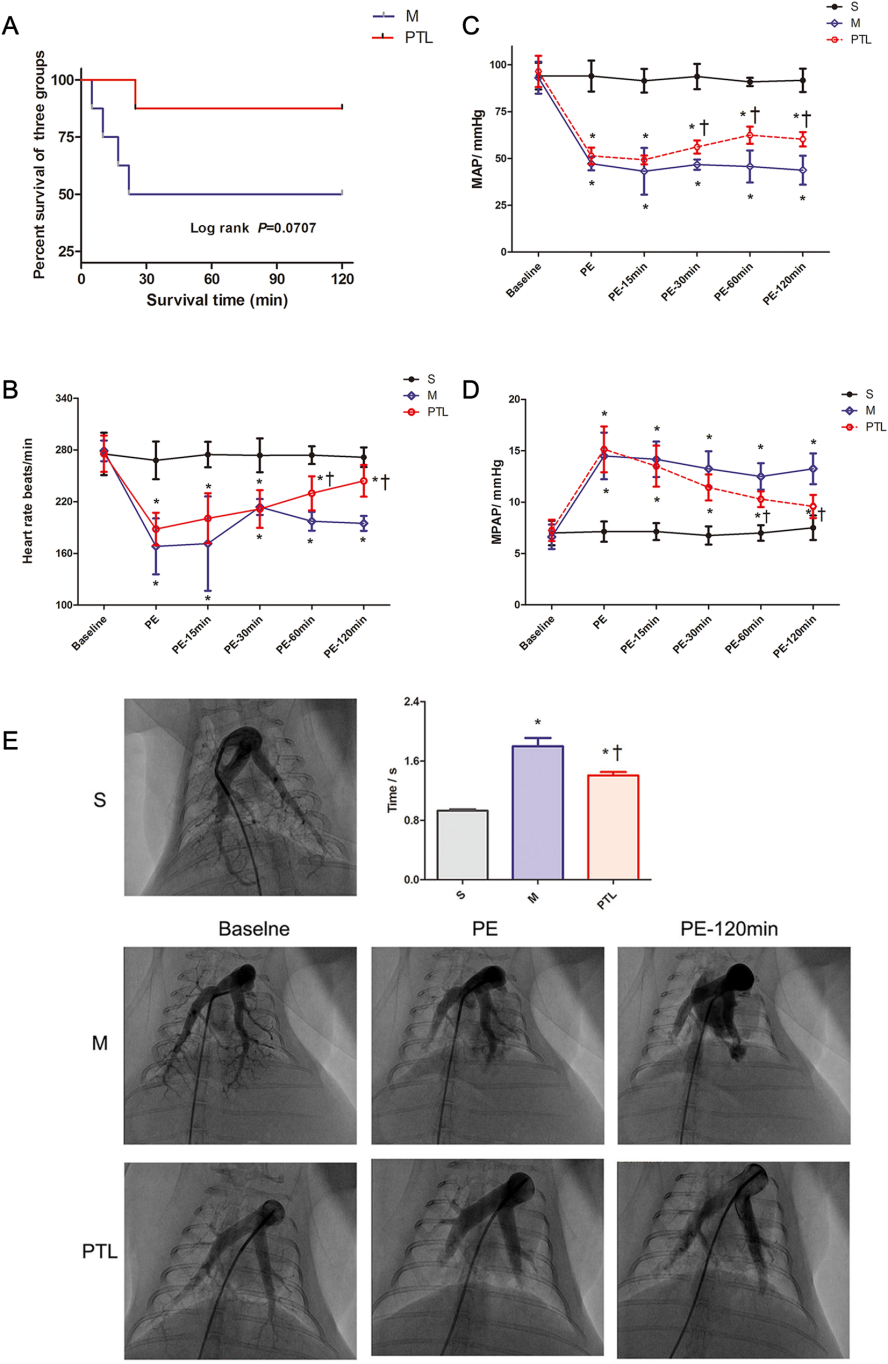
Kaplan–Meier survival curve (**A)**, heart rate (**B)**, average arterial pressure (MAP) (**C)** and average pulmonary arterial pressure (MPAP) (**D)** changes and characteristics of pulmonary circulation (**E)**. S, sham operation group; M, model group; PTL, phentolamine group. Baseline, before pulmonary embolism; PE, pulmonary embolism referring the moment of successful modeling; PE-15 min, 15 min after the moment of successful modeling; PE-30 min, 30 min after the moment of successful modeling; PE-60 min, 60 min after the moment of successful modeling; PE-90 min, 90 min after the moment of successful modeling; PE-120 min, 120 min after the moment of successful modeling.* *P* < 0.05 vs. S group, †*P* < 0.05, vs. M group

As shown in Fig. 1A, all rabbits in S group, 4 rabbits in M group, and 7 rabbits in PTL group survived until the end of the study. 4 rabbits in M group died, with the deaths occurring at 5, 10, 17, 25 min after the moment of successfully modeling, 1 rabbit in PTL group died at 22 min after the moment of modeling.

The rates of shock reversal at 120 min were 0% in the M group and 71.43% in the PTL group. The rate of shock reversal was significantly higher in the PTL group than in the M group (*P* < 0.001).

### Hemodynamics and blood gas changes

As shown in Fig. 1B, the heart rate was significantly reduced at the moment of successful modeling in the M and PTL groups compared to S group, and heart rate tended to recover in both M and PTL groups thereafter, and heart rate of PTL group was similar as S group at 120 min posting successful modeling. After the shock state was induced by rapid injection of autonomous blood clots, MAP of M group and PTL group were significantly decreased, 15 min after at the establishment of successful modeling, the MAP tended to be higher, and was significantly higher at 30 min after the moment of successful modeling in PTL group compared to M group (*P* < 0.05, as seen in Fig. 1C), and remained stable until 120 min after the establishment of successful modeling. As observed in Fig. 1D, MPAP was significantly increased at the moment of successful modeling in both M and PTL group (*P* < 0.05), MPAP tended to be lower at 15 and 30 min after the successful modeling, and was significantly lower at 60 min post modeling in PTL group than in M group.

Oxygenation was measured and hypoxia was evidenced in our acute PE model with shock (Table1). SaO_2_ and PaO_2_ were significantly decreased in M and PTL groups as compared to S group at the moment of successful modeling; whereas, SaO_2_ was significantly higher in PTL group as compared to M group at PE-120 min. PaO_2_ was also significantly higher in PTL group compared to that in M group at PE-120 min. PaCO_2_ was similar among the three groups.

**Table1:** Results of blood gas analysis

Parameter	Time point	S	M	PTL
SaO_2_, %	Baseline	96.75 ± 1.67	96.00 ± 2.00	96.50 ± 1.60
	PE	97.00 ± 1.31	67.88 ± 9.39*	68.38 ± 6.63*
	PE-120 min	97.13 ± 1.36	61.25 ± 6.90*	87.63 ± 5.47*†
PaO_2_, mmHg	Baseline	93.88 ± 4.52	94.50 ± 5.23	95.75 ± 4.03
	PE	93.75 ± 2.92	37.25 ± 5.90*	40.38 ± 5.71*
	PE-120 min	95.50 ± 6.59	47.50 ± 4.04*	70.14 ± 5.64*†
PaCO_2_, mmHg	Baseline	32.38 ± 1.92	33.13 ± 2.23	34.75 ± 3.53
	PE	33.38 ± 1.92	34.50 ± 3.817	34.13 ± 3.56
	PE-120 min	32.00 ± 2.14	35.00 ± 3.56	34.43 ± 3.21

Data are expressed as means ± SD

Baseline before pulmonary embolism, PE pulmonary embolism referring the moment of successful modeling, PE-120 min 120 min after the moment of successful modeling

*S* sham operation group, *M* model group, *PTL* phentolamine group

^*^P < 0.05 vs. S group, †P < 0.05, vs. M group

### Pulmonary arteriography and pulmonary circulation time

As shown in Fig. 1E, pulmonary arteriography was performed at the moment of modeling and at 120 min after the successful modeling, and the pulmonary circulation time of each group was compared. After successful modeling, the pulmonary blood flow in the left and right lower lobes of M group and PTL group were decreased significantly, and the pulmonary circulation time in the two groups were significantly longer than that in S group (*P* < 0.05). At 120 min after successful modeling, pulmonary blood flow in two lung lobes were significantly restored in PTL group as compared to M group, and the pulmonary circulation time was significantly shorter in PTL group than in M group (*P* < 0.05).

### α receptor expression in PE and non-PE tissues

**Fig. 2:**
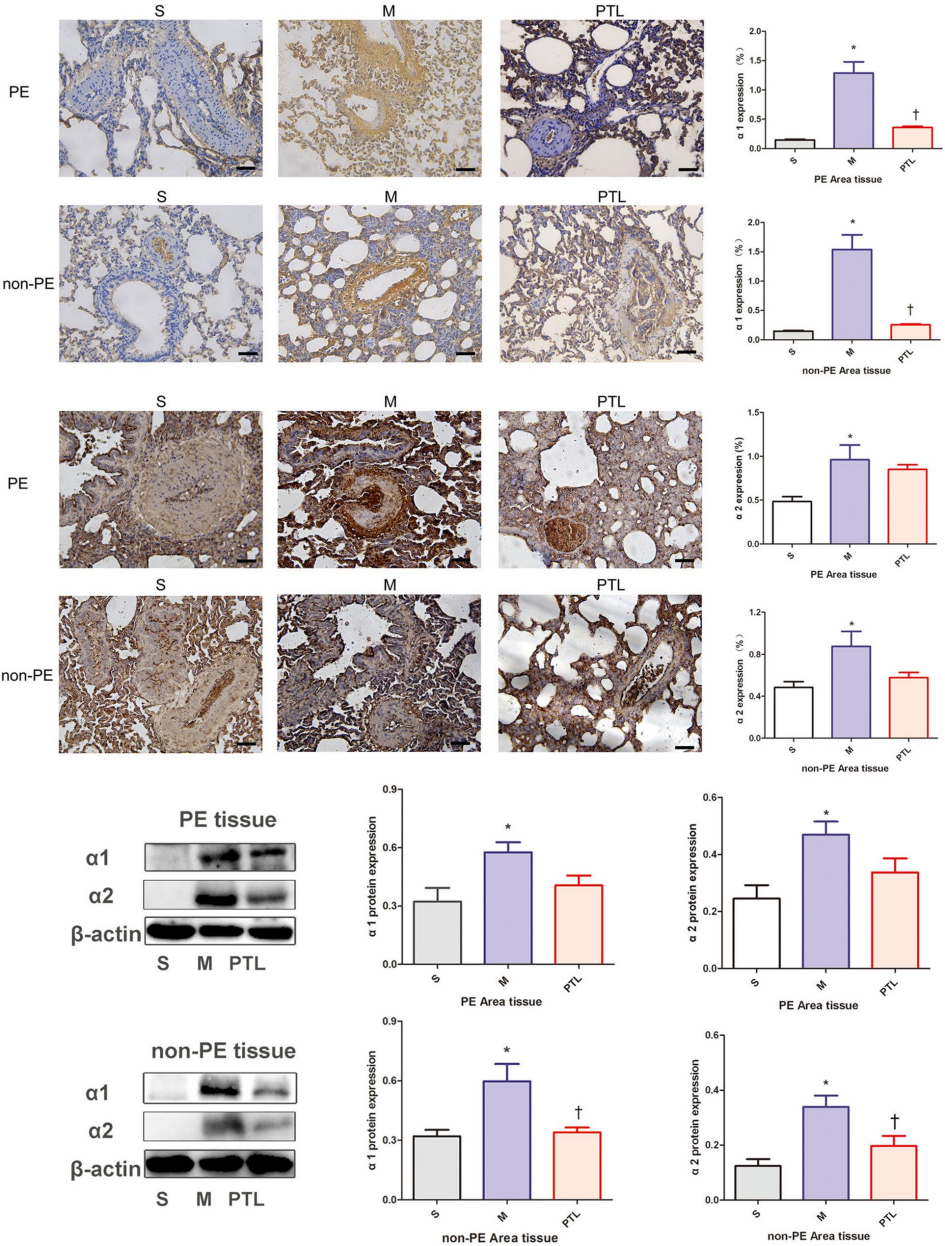
α receptor features of PE and non-PE tissues. S, sham operation group; M, model group; PTL, phentolamine group, PE, pulmonary embolism tissue; non-PE, non-pulmonary embolism tissue. * *P* < 0.05 vs. S group, †*P* < 0.05, vs. M group. Scale bars = 50 µm. Original magnification × 200

α-adrenergic receptors expressed in pulmonary artery smooth muscle cells are involved in regulating the systolic and diastolic activities of pulmonary arteries. The immunohistochemical results are shown in Fig. 2, Additional file 2, in that the expression level of α_1_ receptors in PE and non-PE lung tissues was higher in M group than in S group (*P* < 0.05), and the expression level of α_1_ receptors was lower in PTL group than in M group (*P* < 0.05); the changes of the expression level of α_2_ receptors in PE and non-PE lung tissues had same trend: the expression level of α_2_ receptors in PE and non-PE lung tissues was also higher in M group than in S group (*P* < 0.05), and the expression level of α_2_ receptors was lower in PTL group than in M group (*P* < 0.05).

The results of Western blot showed that in both PE and non-PE lung tissues, the expression levels of α_1_ and α_2_ receptors were higher in M group than in S group (*P* < 0.05), in the non-PE lung tissues, the expression levels of α_1_ and α_2_ receptors were lower in PTL group than in M group (*P* < 0.05), in PE lung tissues, the expression levels of α_1_ and α_2_ receptors showed downward trend in PTL group compared with M group.

### PLC expression in PE and non-PE tissues

**Fig. 3:**
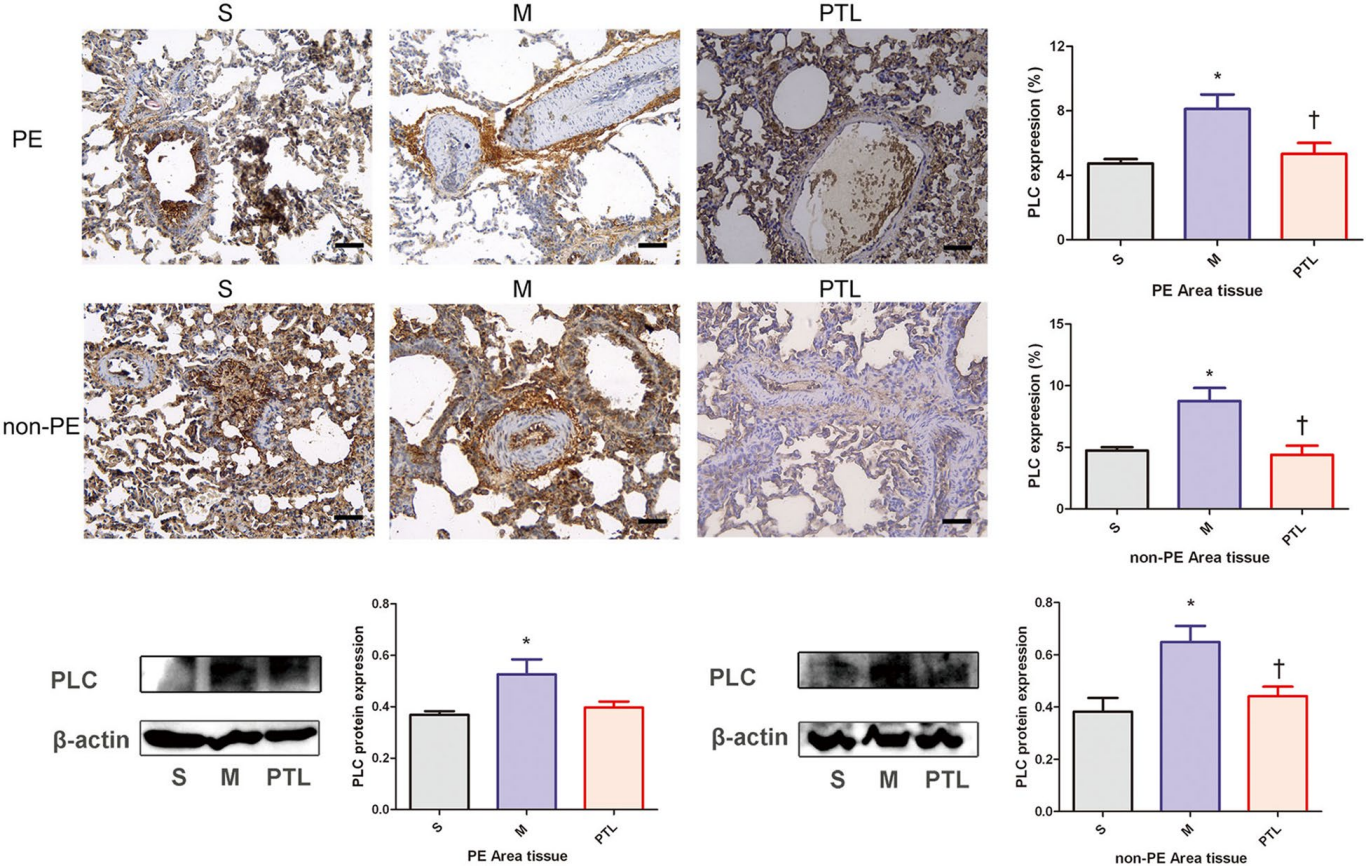
PLC features of PE and non-PE tissues. S, sham operation group; M, model group; PTL, phentolamine group, PE, pulmonary embolism tissue; non-PE, non-pulmonary embolism tissue. PLC, phospholipase C. *P < 0.05 vs. S group, †*P* < 0.05, vs. M group. Scale bars = 50 µm. Original magnification × 200

As shown in Fig. 3, immunohistochemical results demonstrated that PLC expression level was higher in M group compared with that in S group (*P* < 0.05), and PLC expression level was lower in PTL group compared with that in M group in PE and non-PE areas (*P* < 0.05).

As shown in Fig. 3, Western blot results revealed that the expression levels of PLC were significantly higher in M group than in the S group in both the embolized and non-embolized areas (*P* < 0.05); compared with the M group, the expression level of PLC in non-PE lung tissues was significantly down-regulated in PLT group (*P* < 0.05), and the expression level of PLC in PE lung tissues showed a downward trend compared with that in M group.

### t-PKA and p-PKA expression in PE and non-PE tissues

**Fig. 4:**
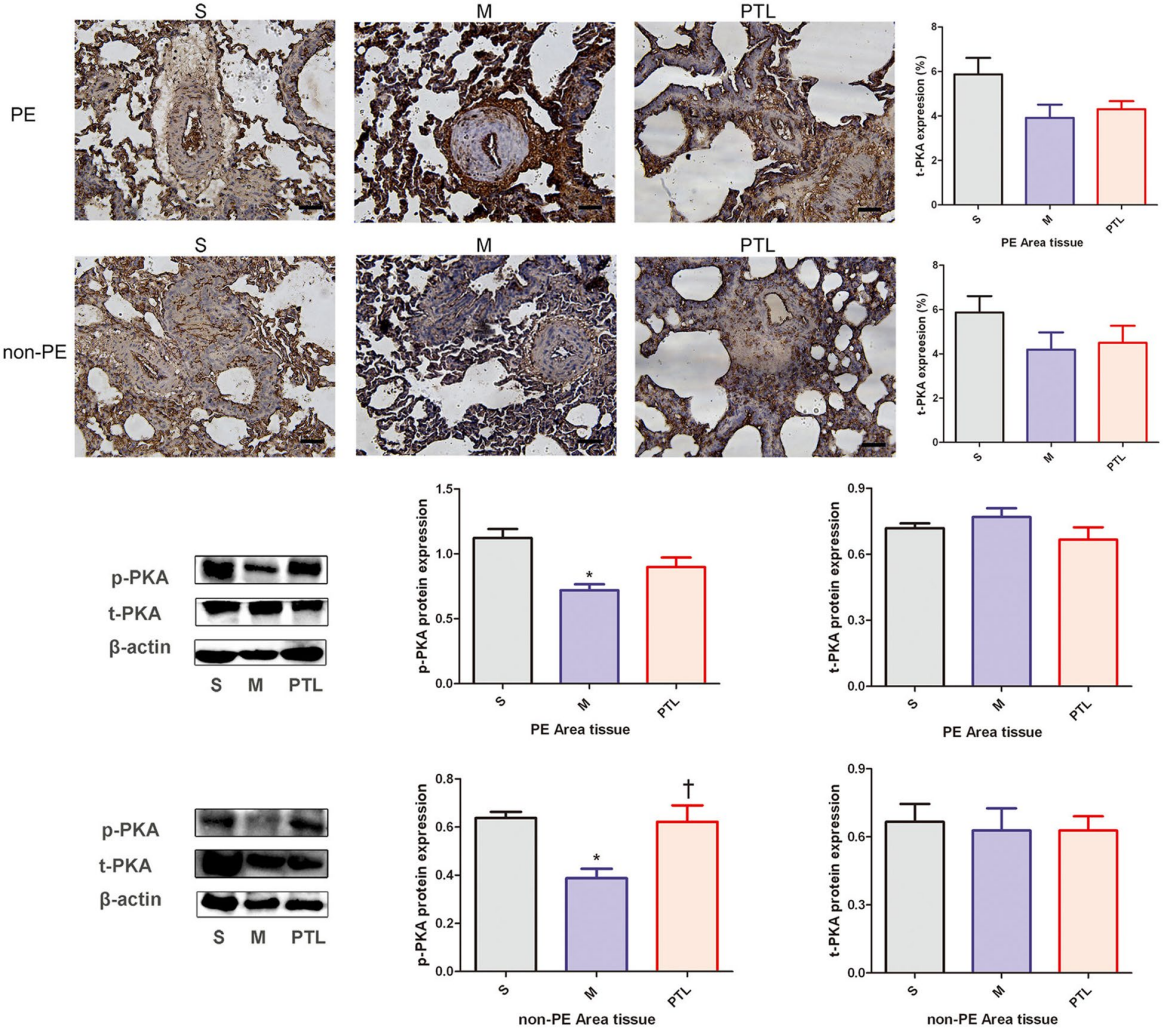
t-PKA and p-PKA features of PE and non-PE tissues. S, sham operation group; M, model group; PTL, phentolamine group, PE, pulmonary embolism tissue; non-PE, non-pulmonary embolism tissue. t-PKA, total protein kinase A, p-PKA, phosphorylated protein kinase A. *P < 0.05 vs. S group, †*P* < 0.05, vs. M group. Scale bars = 50 µm. Original magnification × 200

Immunohistochemistry and Western blot results (Fig. 4) showed that t-PKA expression was similar between S group, M group and PTL group in PE and non-PE lung tissues. In PE and non-PE lung tissues, p-PKA were significantly down-regulated in M group compared with those in S group (*P* < 0.05), and the expression level of p-PKA in non-PE lung tissues was significantly up-regulated in PTL group compared with that in M group (*P* < 0.05), while the expression level of p-PKA in PE lung tissues tended to be up-regulated in PTL group compared with that in M group.

### MLCK expression in PE and non-PE tissues

**Fig. 5:**
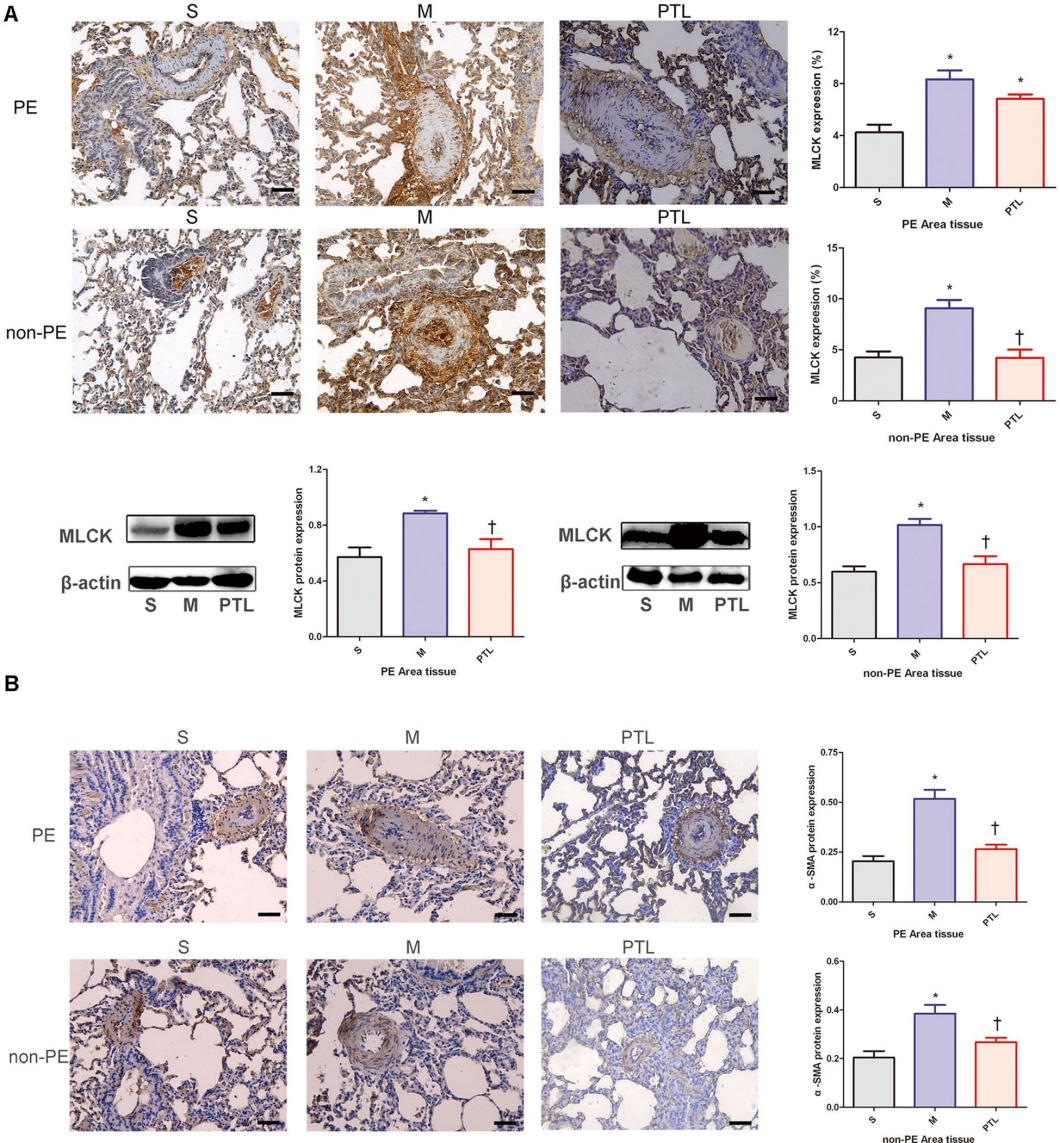
MLCK (**A)** and α-SMA (**B)** features of PE and non-PE tissues. S, sham operation group; M, model group; PTL, phentolamine group, PE, pulmonary embolism tissue; non-PE, non-pulmonary embolism tissue. MCLK, myosin light chain kinase;α-SMA, α-smooth muscle actin.*P < 0.05 vs. S group, †*P* < 0.05, vs. M group. Scale bars = 50 µm. Original magnification × 200

Immunohistochemical results (Fig. 5A) showed that the expression levels of MLCK were higher in the M group than in the S group in both PE and non-PE lung tissues (*P* < 0.05); compared with M group, the expression level of MLCK in the non-PE lung tissues was down-regulated in the PTL group (*P* < 0.05), in the PE lung tissues, the expression level of MLCK trended to be decreased in the PTL group compared with that in M group.

Western blot results (Fig. 5A) suggested that the expression level of MLCK in the non-PE lung tissues and PE lung tissues was up-regulated in the M group compared with that in the S group (*P* < 0.05), and the expression level of MLCK was significantly down-regulated in the PTL group compared with that in the M group (*P* < 0.05).

### α-SMA expression in PE and non-PE lung tissues

α-SMA expressed by pulmonary artery smooth muscle cells is involved in the regulation of pulmonary artery pressure [13]. As shown in Fig. 5B, the expression level of α-SMA in PE and non-PE lung tissues was significantly increased in M group compared with that in S group (*P* < 0.05), and the expression level of α-SMA was significantly decreased in PTL group compared with that in M group (*P* < 0.05).

### Discussion

The major findings of present study are as follows: 1) the activity of α_1_ and α_2_ receptor of pulmonary artery were up-regulated in the non-PE regions lung tissues, activated α_1_ and α_2_ receptor then led to the increase of downstream MLCK expression by regulating PLC and PKA, which collectively induced the pulmonary artery contraction in the setting of APE combined with shock. 2) Bolus intra-pulmonary artery injection of PTL (0.4 mg/kg) could significantly decrease the mean pulmonary artery pressure and improve the pulmonary blood flowing non-PE area, which contribute to the reverse of pulmonary and systemic circulatory failure. 3) PTL significantly down-regulated the expression of α_1_ and α_2_ receptor by inhibiting MLCK activity in the non-PE area. To our best knowledge, this is the first report describing the role and related mechanism of PTL in animal model of APE combined with shock.

### The changes of α receptor activity and its downstream signaling pathways in rabbit model of APE combined with shock

In vascular diseases such as PAH [7–9], the sympathetic system would be activated, followed by enhanced release of catecholamines such as NE, which act at α_1_ receptor to mediate the vasoconstriction process. Sympathetic noradrenergic innervation is widely distributed in rabbit pulmonary blood vessels [14]. Our previous study confirmed that the expression of TH, a mediator related to sympathetic activity, was significantly up-regulated in this model of APE combined with shock [4], TH could catalyze the synthesis and release of NE, and lead to pulmonary vasoconstriction via α receptors on the pulmonary vascular smooth muscle cells [15]. Present study results further revealed that the expression levels of α_1_ receptor were significantly up-regulated in both PE and non-PE lung tissues in this rabbit model of APE combined with shock, synchronous up-regulation of α_1_ receptor expression in non-PE tissues indicated that over-activation of sympathetic activity in the whole lung is one of the main pathological features of APE complicated with shock. Previous research reported, α_1_ receptor could activate the G protein coupled receptor, activate PLC, promote the decomposition of phosphatidyl inositol biphosphate to produce inositol 1,4,5-trisphosphate (IP3), and IP3 then bound to the IP3-sensitive calcium channel in the sarcoplasmic reticulum, which could lead to increased release of Ca^2+^ from the sarcoplasmic reticulum to the cytoplasm [7–9]. Indeed, our results also showed that the expression of PLC in pulmonary artery of non-embolism area was also up-regulated in this model of APE combined with shock, it suggested that, activation of downstream molecule PLC post α_1_ receptor activation could promote the production of IP3. IP3 acted on the IP3-sensitive calcium channel on the sarcoplasmic reticulum in PASMCs, and increase Ca^2+^ release to the cytoplasm, and Ca^2+^ signaling pathway activation further enhance pulmonary vascular constriction in both PE and non-PE lung tissues. At the same time, this study also found that in APE combined with shock model, the expression levels of α_2_ receptor were also significantly up-regulated in both PE and non-PE areas. Previous research reported that α_2_ receptor could activate G protein coupled receptor and inhibit the activation of adenylyl cyclase, reduce the production of cyclic adenosine monophosphate, and then inhibit the activation of PKA [9]. Interestingly, our study also found that p-PKA was substantially decreased in both PE and non-PE lung tissues in this APE combined with shock model. Our results thus suggest that the activation of α_2_ receptor would inhibit PKA, reduce the phosphorylated phospholamban, and further prevent the moving of Ca^2+^ to sarcoplasmic reticulum, accumulated Ca^2+^ in pulmonary artery smooth muscle cells then enhance Ca^2+^ signaling pathway activation post APE.

**Fig. 6:**
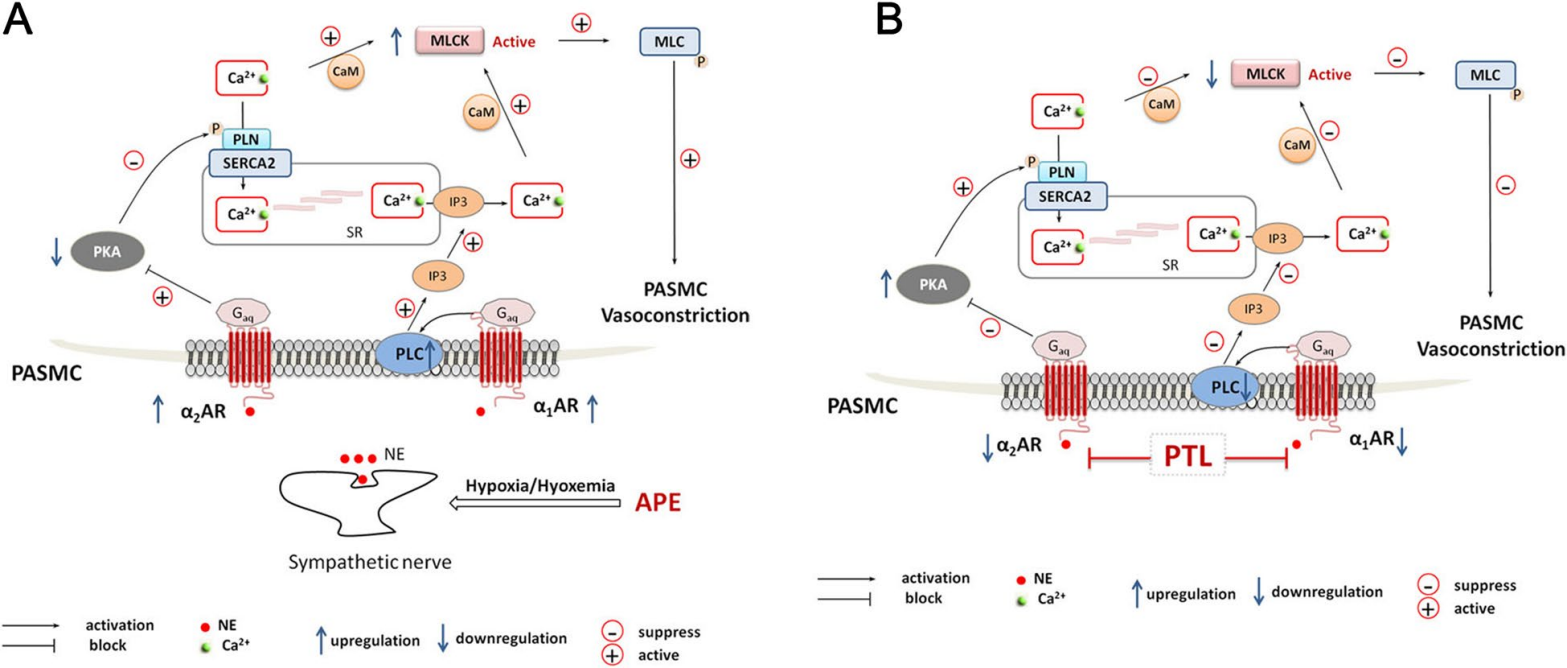
Mechanism of alpha-adrenergic receptor signaling pathway. APE, acute pulmonary embolism; PASMC, pulmonary artery smooth muscle cell; NE, norepinephrine; PLC, phospholipase C; IP3, inositol 1,4,5-trisphosphate; PKA, protein kinase A; SERCA2, sarcoplasmic reticulum Ca^2+^-ATPase; PLN, phospholamban; CaM, calmodulin; MLCK, myosin light chain kinase; MLC, myosin light chain; PTL, phentolamine; SR, sarcoplasmic reticulum

Usually, intracellular free Ca^2+^ combined with calmodulin (CAM) to form a complex to activate MLCK, MLCK mediated the phosphorylation of MLC, and finally stimulated the acceleration of myofilament movement and the contraction of smooth muscle. To determine the mechanism mediating by α_1_ and α_2_ receptors in the model of APE combined with shock, we detected the MLCK expression in PASMCs. As expected, the expression of MLCK was increased in both PE and non-PE areas, which confirmed that the activation of MLCK was involved in the contraction of vascular smooth muscle in this model. This result was consistent with the data of hemodynamics and pulmonary angiography, indicating the attenuation of whole pulmonary blood flow post APE. It is known that acute hypoxia could induce significant increase of sympathetic activity, the up-regulated expression of α_1_ and α_2_ receptor could mediate the contraction of pulmonary artery smooth muscle through Ca^2+^ signaling pathways. Our results hint that the pulmonary artery spasm in non-embolic area played a role in the attenuation of whole pulmonary blood flow and the intracellular Ca^2+^ up-regulation mediated by α_1_ and α_2_ receptor activation in non-embolized lung tissue may serve as one of the main mechanisms of pulmonary vasospasm and pulmonary blood flow loss post APE (as shown in Fig. 6A).

### Hemodynamic effects of PTL treatment on the rabbit model of APE combined with shock

The present study also showed that intra-pulmonary artery ‘bolus’ injection of PTL at the doses of 0.4 mg/kg could reduce the mean arterial pressure, significantly improved the pulmonary blood flow in the non-embolized area, shorten the pulmonary circulation time, reverse pulmonary and systemic circulatory failure in this model, and the results suggest that PTL might be a promising therapeutic option in the treatment of APE combined with shock.

In the case of APE combined with shock, α receptor mediated pulmonary artery contraction may be one of the important causes for the sharp attenuation of whole pulmonary blood flow. Many scholars have carried out related studies regarding the impact of modulating sympathetic activity on acute and chronic PE. Rich and colleagues [16] explored the role of α-adrenergic receptor agonist phenylephrine in PE, they showed that phenylephrine, as a vasoconstrictor, caused a significant increase in MPAP and right ventricular end-diastolic pressure, and a decrease in cardiac output and pulmonary artery oxygen saturation in patients with pulmonary hypertension. Meanwhile, in experimental canine pulmonary embolism model, Hirsch and coworkers found that phenylephrine treatment increased MPAP and decreased cardiac output [17]. Above results thus indicate that phenylephrine could increase MPAP but at the cost of reducing cardiac output, which might worsen the shock status post acute PE. For this reason, the effects of phenylephrine were not tested in our acute PE model with cardiac shock.

Lyhne and colleagues [18] evaluated the effects of levosimendan, milrinone, and dobutamine in a porcine acute pulmonary embolism model, results showed that levosimendan and milrinone reduced right ventricular afterload and improved right ventricular function, whereas dobutamine at higher doses increased right ventricular afterload and right ventricular mechanical work. Future studies are warranted to observe the impact of above drugs in our acute PE model with shock.

It is of great significance to find an effective intervention method to expand pulmonary artery, alleviate pulmonary circulation failure, promote the recovery of peripheral blood pressure and reverse shock. Previous reports documented that inhaled nitric oxide (NO) treatment could dilate the pulmonary vasculature after acute PE. However, the results of clinical studies showed that the clinical symptoms, ventricular function and prognosis of patients were not significantly improved [19, 20]. Theoretically, inhaled NO traveled through the alveoli and pulmonary capillaries and then to the pulmonary vein, but not pulmonary artery. The role of inhaled NO might thus be limited in our acute PE with shock model.

Another clinical study compared the efficacy of intravenous SNP and inhaled NO in the treatment of pulmonary hypertension, and found that the effect of controlled intravenous SNP infusion was better than that of NO inhalation for the reduction of MPAP and PVR in this clinical setting [21]. Our previous experiment also investigated the effect of SNP on the rabbit acute PE with shock. Results showed that SNP with dose titration strategy could effectively dilate pulmonary arteries, attenuate the vasoconstrictive responses to acute PE, improved pulmonary blood flow in non-PE areas and reversed systemic shock [5].

In the acute PE model with shock, pulmonary blood flow and systemic circulation failure could activate sympathetic activity, and then induce the over-activation of sympathetic activity and further aggravate pulmonary artery spasm in PE and non-PE regions, thus forming a vicious cycle. Our previous study showed SNP treatment could reduce the expression of sympathetic mediators in the lung tissue, which might be the result of the reduction of hypoxemia, pulmonary vasculature spasm and sympathetic activation, not through the direct effect of SNP on sympathetic activity [4]. In addition, since SNP had a short half-life and limited duration of action, it is necessary to find a drug that could continuously reduce sympathetic activity and relieve pulmonary artery spasm in the setting of acute PE and shock.

In previous study [22], carvedilol was applied in isolated perfused lungs in the rabbit model of pulmonary thromboembolism to observe its effects on pulmonary microcirculation, the results showed that carvedilol treatment could decrease precapillary resistance and pre/postcapillary resistance ratio, and probably by acting with α receptor. In another study [23], PTL effectively improved pulmonary arterial pressure and pulmonary vascular resistance in experimental model of pulmonary thromboembolism. Although α receptor blockers used in the pulmonary artery and PE model had achieved significant beneficial results, none of these studies observed the impact of α receptor blockers in the setting of APE with circulatory shock, in fact, α receptor blockers can dilate pulmonary arteries, and at the same time, it can also dilate vessels of the systemic circulation. To achieve selective dilation of pulmonary arteries and reduction of pulmonary artery pressure without reducing peripheral pressure, and avoid the deterioration of shock, we need carefully choose the drug administration route and dosage. Therefore, we investigated the intervention effect of PTL in our model at various dosages in the pilot study.

It is known that the plasma concentration of PTL peaked in about 2 min after iv injection, and the effect could last 15–30 min. In previous studies [24], the peripheral injection of 0.5 mg/kg PTL did not significantly affect MAP in rats, and the administration of 1 mg/kg PTL selectively dilated the renal artery, and had no obvious effect on other vessels. After the successful modeling of APE complicated with shock, peripheral MAP of rabbits was significantly decreased post the injection of 0.5–1.0 mg/kg PTL via the pulmonary artery. Further experiments showed that peripheral MAP increased gradually when PTL was administered via pulmonary artery at the dosage of 0.1 mg/kg and 0.4 mg/kg. In the main study, PTL was bolus injected via pulmonary artery at the doses of 0.4 mg/kg. Experimental results showed that pulmonary circulation and systemic circulation began to improve at 15 min after drug administration. Systemic circulation improved significantly at 30 min after drug administration, and the effects remained up to 120 min. Our results thus demonstrated that bolus pulmonary artery injection of low-dose PTL could block the up-regulation of sympathetic activity in the whole lung, mediate pulmonary artery dilation in PE and non-PE tissues, and improve pulmonary blood flow, therefore reducing MPAP and increasing MAP, which might be an effective therapy option for the treatment of APE complicated with shock. Meanwhile, these features show that PLT might have translational value in future clinical practice. Future clinical studies are warranted to evaluate the effects of PLT in patients with acute PE and shock.

### The effect of PTL on α receptors and their downstream signaling pathways in the whole lung tissue

To explore the underlying mechanisms, we further observed the expression of α receptor in whole lung tissues after PTL intervention. After PTL treatment, the expression of α_1_ receptor and PLC were significantly down-regulated in both PE and non-PE tissues, this suggests that PTL blocked α_1_ receptor-related signaling pathway, PLC inhibition would further reduce the production of IP3, Ca^2+^ released from sarcoplasmic reticulum and the concentration of Ca^2+^ in PASMCs. Meanwhile, α_2_ receptor-related signaling pathway was also inhibited in both PE and non-PE tissues, the expression of α_2_ receptor were down-regulated, p-PKA was up-regulated, indicating that PTL could also down-regulate α_2_ receptor and increase the activation of p-PKA, promote the phosphorylation of phospholamban, Ca^2+^ moving into sarcoplasmic reticulum, and leading to further reduced Ca^2+^ concentration in PASMCs. Taken together, PTL can down-regulate the expression of α_1_ and α_2_ receptor, inhibit its downstream signal pathway, and finally inhibit Ca^2+^ signaling pathway. Intracellular complex of free Ca^2+^ and CAM will decrease, MLCK activation will be inhibited, myofilament movement will slow down and leading to vascular relaxation in this rabbit model of APE complicated with shock. Post PTL, the expression of MLCK and α-SMA were down-regulated in both PE and non-PE lung tissues, further confirmed that PTL could dilate pulmonary artery and reduce pulmonary vascular resistance by inhibiting α receptor activity and its downstream signaling pathway. Down-regulating intracellular Ca^2+^ by inhibiting α_1_ and α_2_ receptor activity activated by sympathetic nerve in non-embolized lung tissues may be the main mechanisms of PTL on dilating pulmonary arteries and improving the pulmonary blood flow in this model (Fig. 6B).

It is noteworthy that PTL treatment showed the same trend of α receptors and their downstream signaling molecules in PE and non-PE lung regions, but there were still expression differences between the two lung tissues. Western blot results showed that compared with the M group, the changes of α_1_ receptor, PLC, α_2_ receptor and p-PKA in the non-PE lung region were statistically significant, but not statistically significant in the PE lung region. Immunohistochemical results showed that compared with M group, MLCK and p-PKA in PTL group were significantly different in non-PE lung tissues, and there was only a trend of change in PE tissues. This prompted that compared with PE tissues, the intervention effect of PTL in non-PE tissues was greater than in PE tissues. This may be explained as follows: the main reason for blood flow loss in non-PE tissues is pulmonary vascular spasm, rather than mechanical obstruction. As the pulmonary artery spasm could be gradually improved after PTL treatment, but PTL was unable to combat the mechanical obstruction in the PE lung tissues.

### Study limitation

It is to note that due to study design deficit, we did not perform echocardiography examination in our study, echocardiography-derived cardiac output, right ventricular function parameters like TAPSE and RVOT VTI might provide further information on the impact of PTL in this model. Future studies are needed to clarify this issue.

### Conclusions

The activation of α_1_ and α_2_ receptor and its downstream signaling pathway in APE could induce pulmonary spasm, which is responsible for the rapid decline of blood flow in non-PE lung tissues, and the exacerbation of pulmonary hypertension, right heart failure and systemic circulatory shock. PTL treatment could effectively down-regulate α receptor activity and block its downstream signaling pathway, and relieve pulmonary artery spasm, especially in the non-embolization area, pulmonary blood flow recovery in the non-embolization area could then effectively break the pathophysiological vicious cycle of APE combined with shock, and reverse pulmonary and systemic circulatory failure in this rabbit model of APE combined with shock. Future studies are warranted to validate the translational impact of applying bolus pulmonary artery PTL injection for patients with APE combined with shock.

## Supplementary Information

**Additional file 1: Figure S1. **Effects of PTL dose findings experiments on average arterial pressure (MAP), average pulmonary arterial pressure (MPAP) and heart rate were shown in Sham rabbits and acute PE rabbits.

**Additional file 2.** The complete Western blot figures.

## Data Availability

The datasets generated during the current study are available from the corresponding author on reasonable request.

## Abbreviations

APE: Acute pulmonary embolism
PTL: Phentolamine
MPAP: Mean pulmonary arterial pressure
MAP: Mean arterial pressure
PE: Pulmonary embolism
non-PE: Non-pulmonary embolism
PLC: Phospholipase C
p-PKA: Phosphorylated protein kinase A
MLCK: Myosin light chain kinase
MLC: Myosin light chain
α-SMA: α-Smooth muscle actin
TH: Tyrosine hydroxylase
NE: Norepinephrine
IP3: Inositol 1,4,5-trisphosphate
t-PKA: Total protein kinase A
CAM: Calmodulin
