# Supplementary figures and images for: Effects and related mechanism of alpha-adrenergic receptor inhibitor phentolamine in a rabbit model of acute pulmonary embolism combined with shock
Source: Eur J Med Res. 2022 Nov 8;27:238. doi: 10.1186/s40001-022-00842-5 (PMC9641939; doi:10.1186/s40001-022-00842-5)

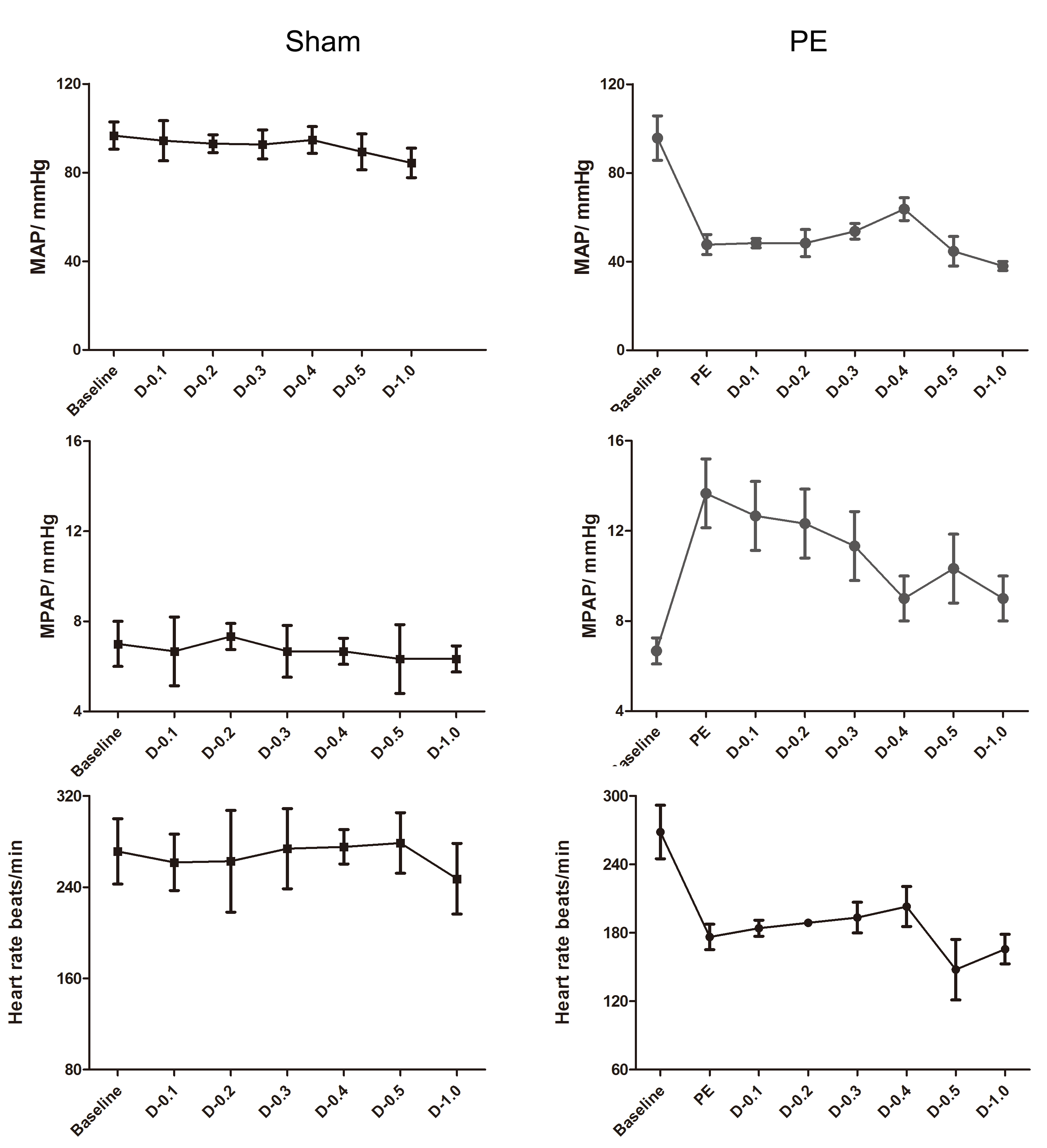

Supplement: Supplementary file 1 — Additional file 1: Figure S1. Effects of PTL dose findings experiments on average arterial pressure (MAP), average pulmonary arterial pressure (MPAP) and heart rate were shown in Sham rabbits and acute PE rabbits. [file 40001_2022_842_MOESM1_ESM.tif]

Supplementary Figure


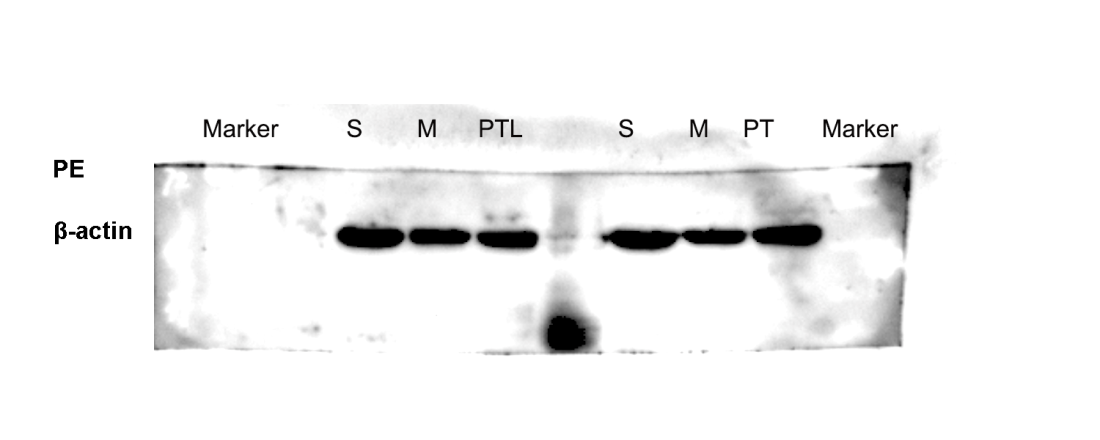


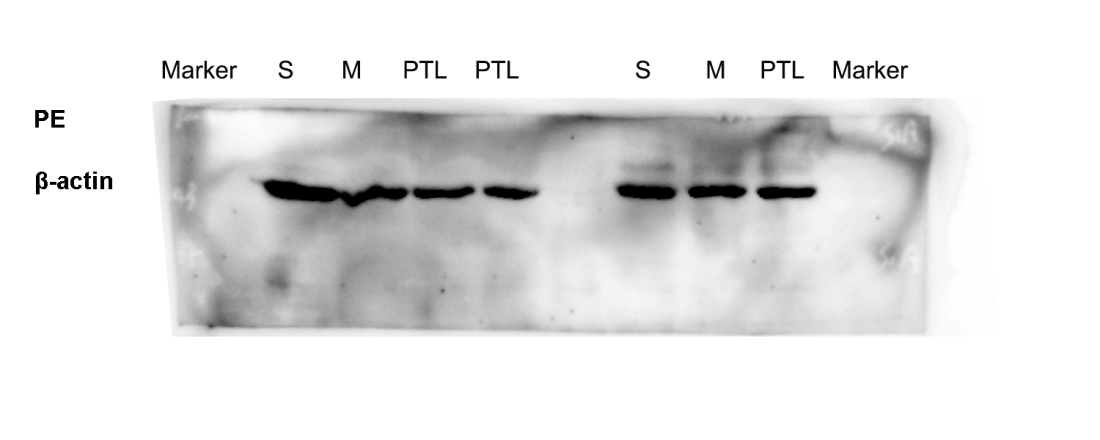


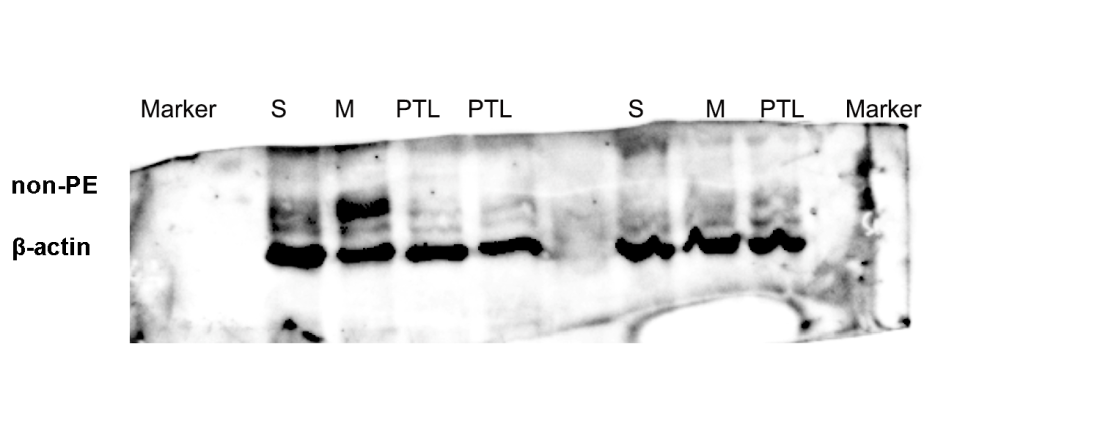


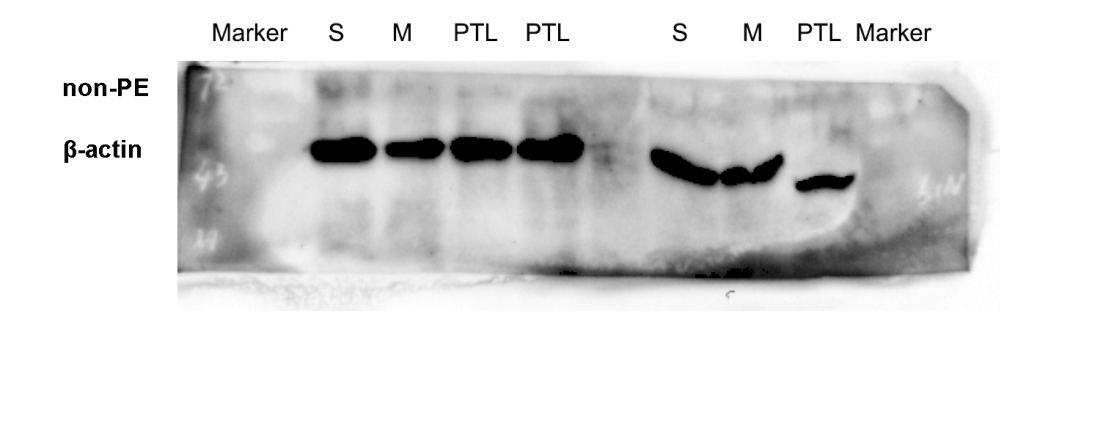


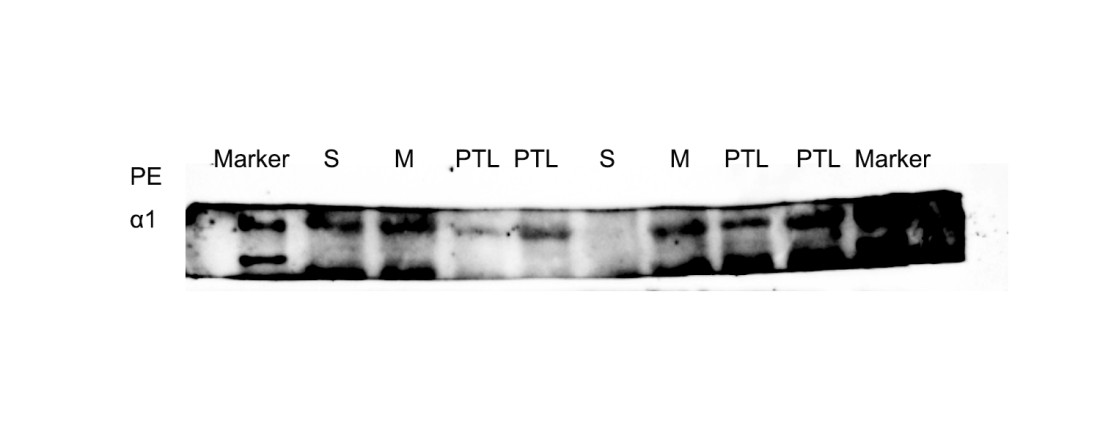


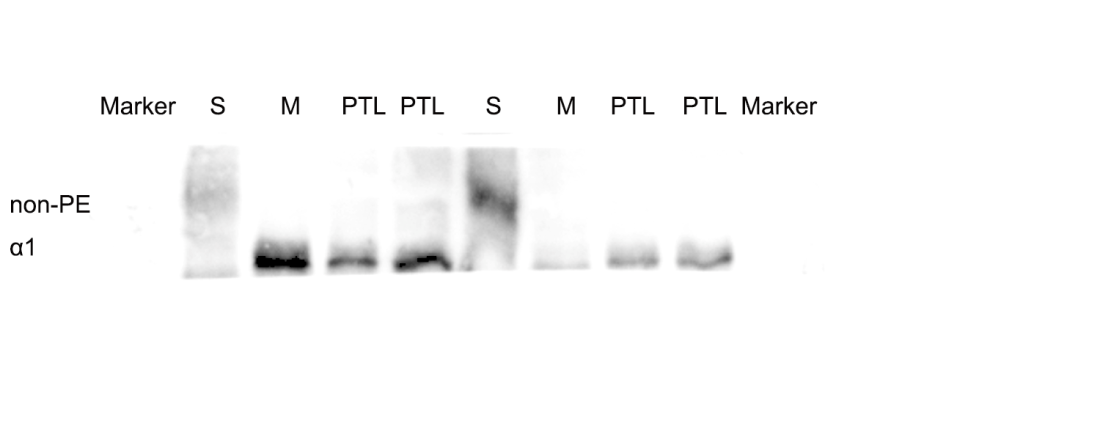


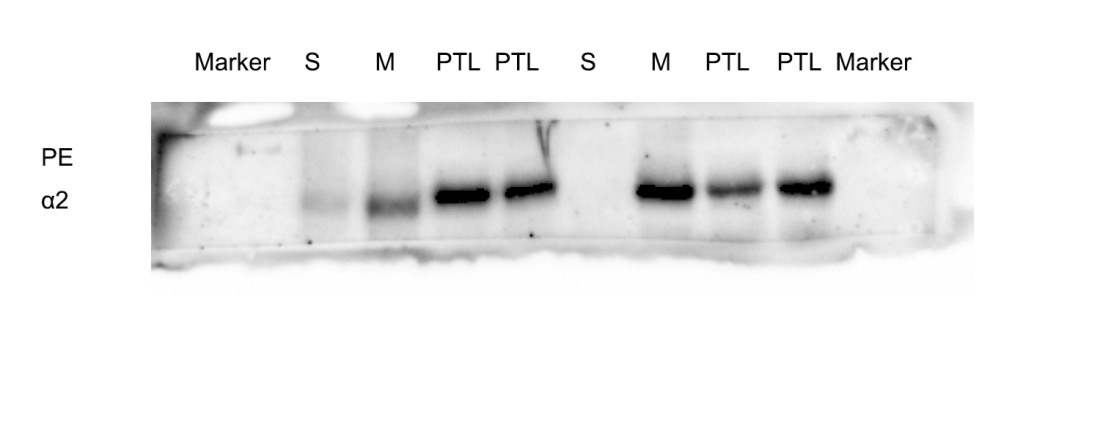


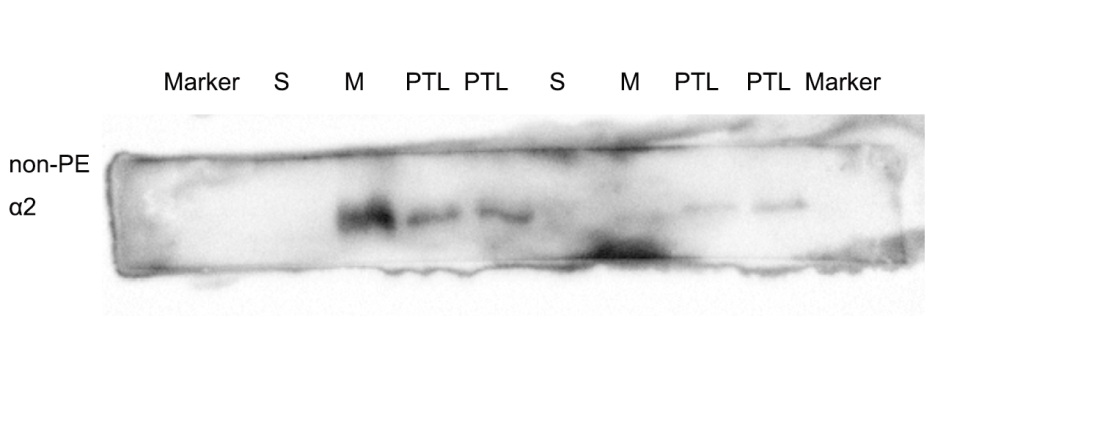


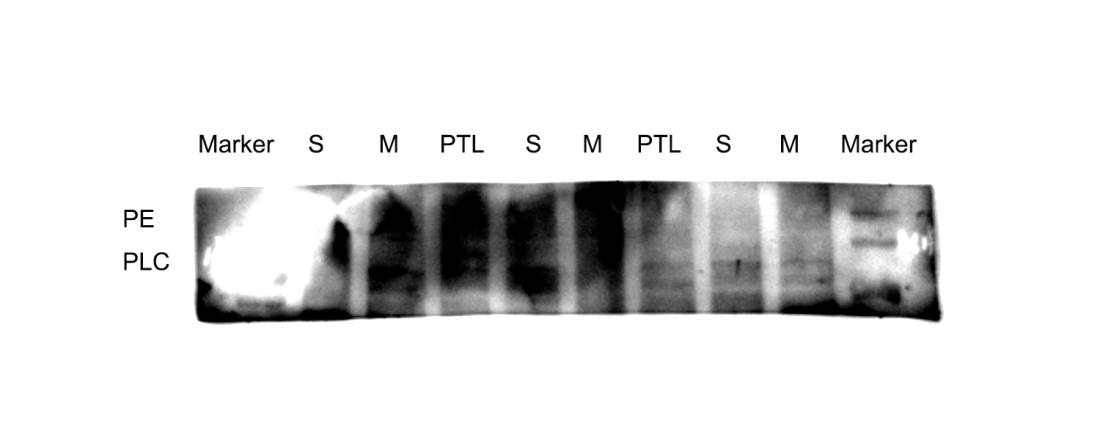


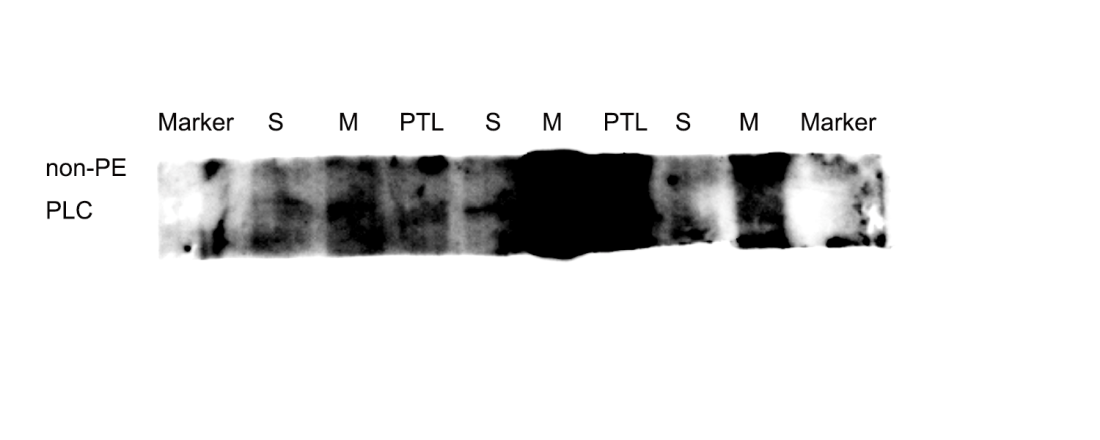


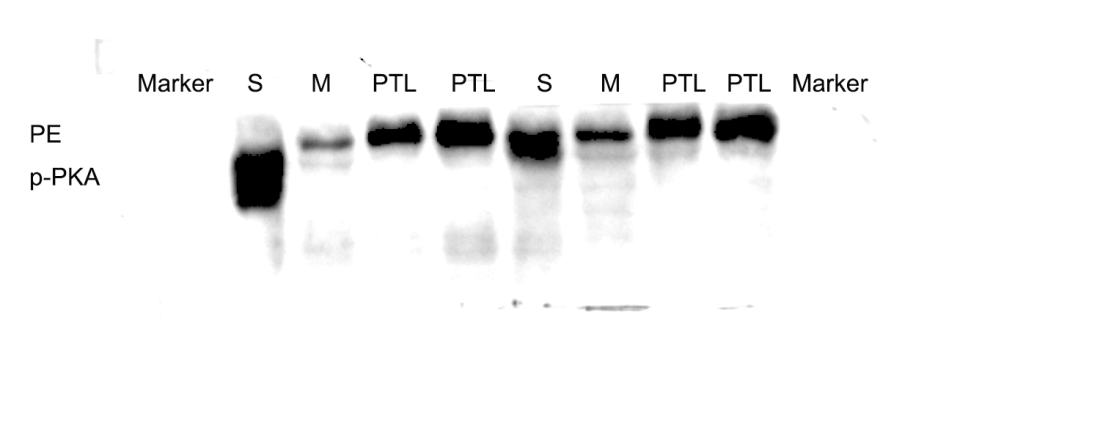


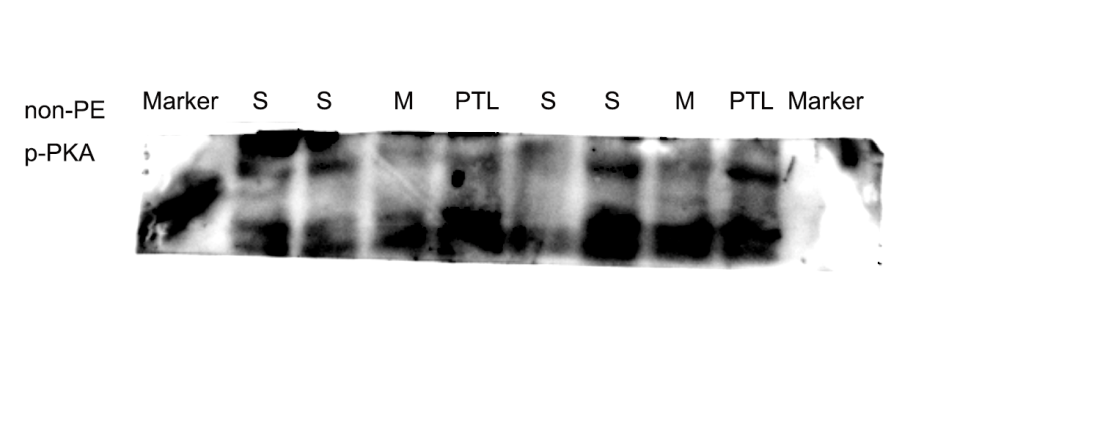


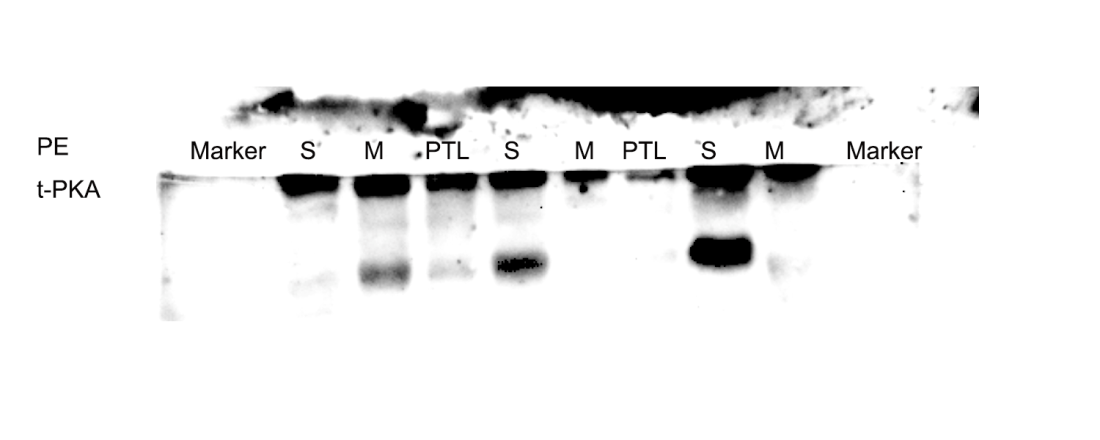


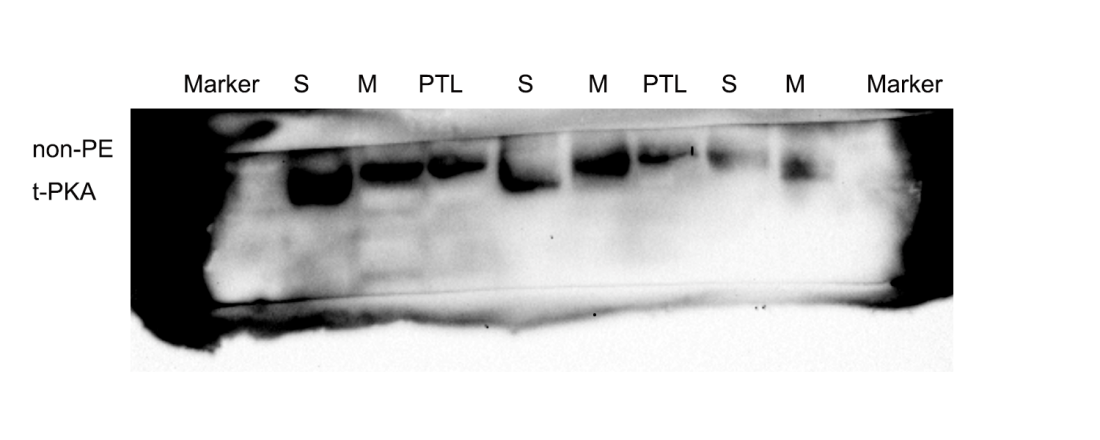


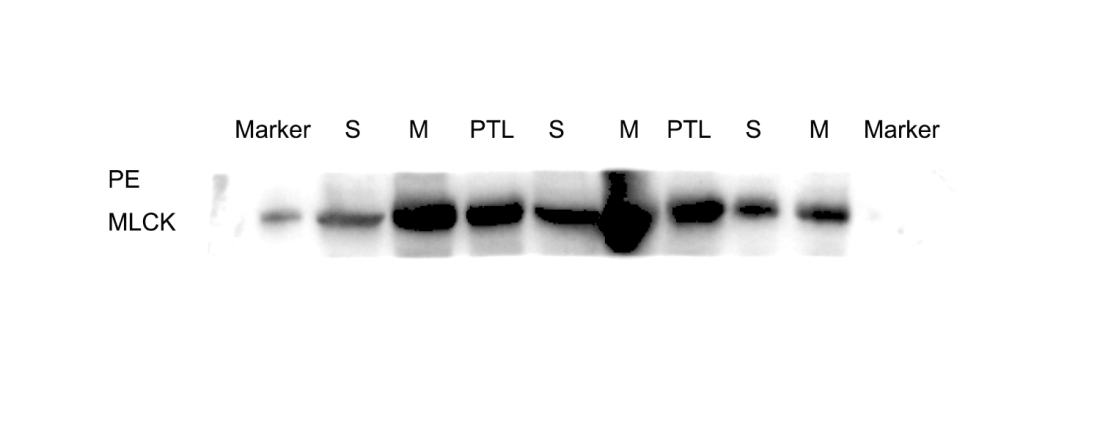


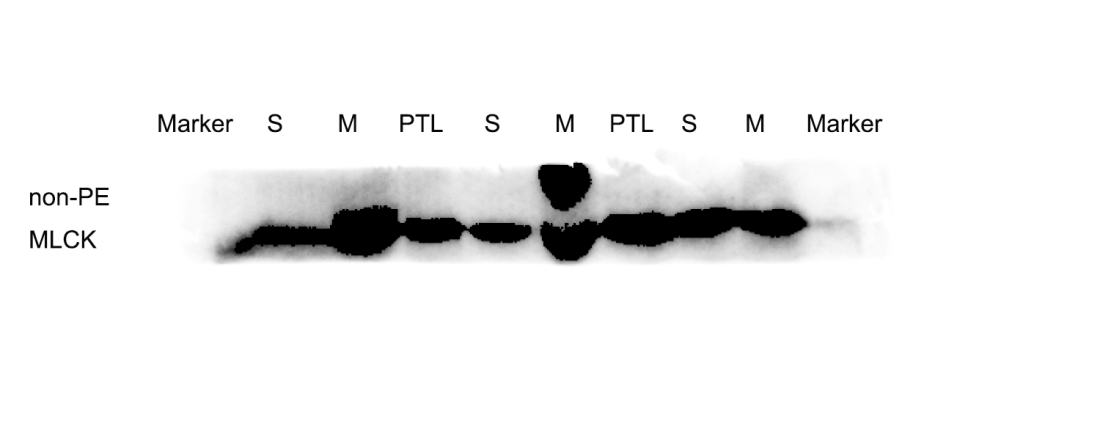

Supplement: Supplementary file 2 — Additional file 2. The complete Western blot figures. [file 40001_2022_842_MOESM2_ESM.docx]
